# Mycoplasma Chromosomal Transfer: A Distributive, Conjugative Process Creating an Infinite Variety of Mosaic Genomes

**DOI:** 10.3389/fmicb.2019.02441

**Published:** 2019-10-23

**Authors:** Emilie Dordet-Frisoni, Marion Faucher, Eveline Sagné, Eric Baranowski, Florence Tardy, Laurent Xavier Nouvel, Christine Citti

**Affiliations:** ^1^IHAP, INRA, ENVT, Université de Toulouse, Toulouse, France; ^2^UMR Mycoplasmoses des Ruminants, VetAgro Sup, Laboratoire de Lyon, ANSES, Université de Lyon, Marcy-l’Étoile, France

**Keywords:** mycoplasmas, horizontal gene transfer, conjugation, chromosomal transfer, evolution

## Abstract

The capacity of Mycoplasmas to engage in horizontal gene transfers has recently been highlighted. Despite their small genome, some of these wall-less bacteria are able to exchange multiple, large portions of their chromosome via a conjugative mechanism that does not conform to canonical Hfr/*oriT* models. To understand the exact features underlying mycoplasma chromosomal transfer (MCT), extensive genomic analyses were performed at the nucleotide level, using individual mating progenies derived from our model organism, *Mycoplasma agalactiae*. Genome reconstruction showed that MCT resulted in the distributive transfer of multiple chromosomal DNA fragments and generated progenies composed of a variety of mosaic genomes, each being unique. Analyses of macro- and micro-events resulting from MCT revealed that the vast majority of the acquired fragments were unrelated and co-transferred independently from the selection marker, these resulted in up to 17% of the genome being exchanged. Housekeeping and accessory genes were equally affected by MCT, with up to 35 CDSs being gained or lost. This efficient HGT process also created a number of chimeric genes and genetic micro-variations that may impact gene regulation and/or expression. Our study unraveled the tremendous plasticity of *M. agalactiae* genome and point toward MCT as a major player in diversification and adaptation to changing environments, offering a significant advantage to this minimal pathogen.

## Introduction

The sexual reproduction of eukaryotic organisms is a powerful mechanism of genetic diversity that eliminates deleterious mutations and results in a higher quality of offspring (Hörandl, 2009). In bacteria, one main driver of evolution and diversification is provided by horizontal gene transfer (HGT; Soucy et al., 2015; García-Aljaro et al., 2017). When compared to eukaryotic meiosis, this process often produces much less genetic mixing and mainly concerns the spread of mobile accessory genes. Yet, HGT is key in allowing bacteria to adapt to a variety of specific niches or conditions via the acquisition of genes encoding specific metabolic pathways, toxins or, for instance, proteins conferring resistance to antibiotics (Ochman et al., 2000; Soucy et al., 2015).

Bacteria have evolved a number of strategies to perform HGT that rely or not on mobile genetic elements (Frost et al., 2005; García-Aljaro et al., 2017). The three primary modes of HGTs are transformation, transduction and conjugation, the latter being considered as predominant and responsible for the rapid dissemination and acquisition of new, adaptive traits upon cell-to-cell contact (Frost and Koraimann, 2010; Halary et al., 2010). Conjugation traditionally required the donor bacterial cell to express (i) a “conjugative pilus” or a multicomponent membrane traversing structure, also known as the “conjugative pore” that promotes and maintains cellular contact of the mating-pair, and (ii) a conjugative machinery responsible for DNA transfer into the recipient cell, via the conjugative pore and upon recognition of an origin of transfer (oriT) (Goessweiner-Mohr et al., 2014). This two-step process is usually mediated by plasmids or integrative and conjugative elements (ICE) and mainly concerns the transfer of mobile genetic elements themselves. Yet, some conjugative mechanisms can also involve the transfer of chromosomal DNA regions as initially described for the Hfr system of *Escherichia coli* (Wollman et al., 1956). There, Hfr- or *oriT*-chromosomal transfers are initiated from an *oriT* located in the genome, and are characterized by a gradient, with genes closer to the *oriT* being more frequently transferred and thus incorporated into the recipient chromosome (Smith, 1991). Recently, our group discovered a novel form of chromosomal conjugative transfer occurring in mycoplasmas that did not conform to classic *oriT*-based models (Dordet-Frisoni et al., 2014; Faucher et al., 2019).

With over 200 species, mycoplasmas represent a large group of bacteria that are characterized by the lack of a cell wall and a small-size genome (580–2,200 kb) (Razin and Hayflick, 2010). Despite this simplicity, numerous mycoplasma species are important pathogens of man and animals (Razin and Hayflick, 2010). These bacteria derived from Gram-positive ancestors and their evolution was long considered as degenerative, with successive losses of genetic materials (Sirand-Pugnet et al., 2007). This paradigm was initially based on the mycoplasma paucity in recombination systems, phages or conjugative elements, but has been challenged over the past years by comparative genomic studies (Sirand-Pugnet et al., 2007). Indeed, the genome of several *Mycoplasma* spp. phylogenetically remote but sharing the same habitat has been shaped by large DNA exchanges (Vasconcelos et al., 2005; Sirand-Pugnet et al., 2007; Pereyre et al., 2009) and ICEs were detected in a large number of sequenced mycoplasma genomes (Calcutt et al., 2002; Marenda et al., 2006; Dordet-Frisoni et al., 2013; Tardy et al., 2015), raising the prospect that these simple bacteria might be able to conjugate.

Our group highlighted two conjugative processes occurring among and within certain *Mycoplasma agalactiae* (Ma) strains, an important ruminant pathogen, and a model organism. The first process is the conventional horizontal dissemination of a mycoplasma ICE (MICE), from ICE-positive to ICE-negative cells (Dordet-Frisoni et al., 2013). MICEs are a new family of large modular elements that encode the machinery for their self-transmission and maintenance including conjugation, excision and integration into the chromosome of the recipient cell. MICE transfer occurs following a clean cut and paste mechanism and is not accompanied by the transfer of flanking chromosomal sequences (Dordet-Frisoni et al., 2013). The second concerns the transfer of large blocks of chromosomal DNA from ICE-negative to ICE-positive cells and was further referred to as mycoplasma chromosomal transfer (MCT) (Dordet-Frisoni et al., 2014). MCT is an atypical mechanism of HGT and is not directly linked to ICE- or other mobile genetic elements-movements, but relies on MICE conjugative properties: for MCT to occur, at least one mating partner must carry a functional MICE (Dordet-Frisoni et al., 2013; Baranowski et al., 2018). MCTs result in replacing large chromosomal regions of the recipient cell by the donor-counterparts via recombination (Dordet-Frisoni et al., 2014). Whole genome sequencing of populations obtained after *in vitro* mating revealed that these DNA exchanges could equally affect any part of the donor chromosome and differ from classical *oriT*-based mechanisms (Dordet-Frisoni et al., 2014). More recently, DNase-resistant HGT events have been reported in the human pathogen *Mycoplasma genitalium* that depended on RecA homologous recombination (Torres-Puig et al., 2018) and further investigations are needed to define whether the outcomes of this conjugative process are similar to that observed in *M. agalactiae*. So far no mobile genetic element such as ICE has been identified in the genome of *M. genitalium.*

In the past 10 years, the emergence of new pathogens with mosaic-like genomes (Kloesges et al., 2011; Lesic et al., 2012; Watt et al., 2018) led to the discovery of new chromosomal conjugative transfer mechanisms also diverging from the classical Hfr or *oriT*- based model. For instance, a new conjugation-like mechanism termed transjugation has been described in *Thermus thermophilus* that involves a “*push-pull*” system in which the donor cell actively pushes out chromosomal or plasmid DNA while the recipient cell exploits its natural competence system to uptake genomic DNA (Blesa et al., 2017). Another atypical conjugative mechanism has been found in *Yersinia pseudotuberculosis*, where the presence of an IS*6*-type element on a conjugative replicon, is able to convey the transfer of virtually any piece of chromosomal or plasmid DNA (Lesic et al., 2012). Finally, the distributive conjugal transfer of *Mycobacterium smegmatis* is one of the most documented and fascinating mechanisms of unconventional, conjugative, chromosomal transfer (Gray et al., 2013; Derbyshire and Gray, 2014; Gray and Derbyshire, 2018). This mechanism promotes the distributive transfer of multiple, non-contiguous genome segments from a donor to a recipient. As observed for MCT, the exchange of donor chromosome occurs with equal efficiency regardless of their location. Distributive conjugal transfer is a chromosomally encoded process that leads to the genetic mixing of two parental bacteria generating progenies with highly mosaic genomes (Gray et al., 2013).

Our initial description of MCT was mainly based on genomic analyses of mixed clonal populations (Dordet-Frisoni et al., 2014). From these data, we showed that the entire genome was mobile and hypothesized that one to two large genomic segments could be transferred during a single mating experiment. Recently, our group highlighted the role of MCT in the acquisition and dissemination of antibiotic resistance (Faucher et al., 2019). During that study, whole genome sequencing of several individual transconjugants revealed that MCT was more complex than we first thought, with offspring that simultaneously inherited multiple distant and unrelated fragments (Faucher et al., 2019). These two extreme situations raised the question of the general features defining MCT and of the forces that influence the degree of genome mosaicism. We addressed these issues using a set of single transconjugants that derived from different mating pairs in which the position, nature and type of selectable marker varied. Mosaic genomes were observed in all progenies, regardless of the conditions applied, with up to 17% of the total size of the donor genome being transferred. Data also indicated that the incorporation of donor DNA in the host chromosome relied on both homologous and non-homologous recombination mechanisms that were independent from the ICE conjugative machinery. Overall, MCT was shown to result in creating both macro- and micro-complexity, affecting housekeeping genes and accessory genes equally. Our data indicate that the environmental conditions such as the use of fluoroquinolone may affect the level of genome mosaicism. We further discussed how the extraordinary plasticity of the mycoplasma small-size genome may impact its evolution and promote adaptation to new environments.

## Materials and Methods

### Mycoplasma Strains and Culture Conditions

*Mycoplasma. agalactiae* (Ma) strains PG2, 5632, 14628, 4055, and 4867 were used in this study (Tables 1, 2 and Supplementary Table S1). Mycoplasmas were grown at 37°C in SP4 medium supplemented with cefquinome (45 μg mL^–1^, cobactan, MSD Animal Health), and with gentamicin (50 μg mL^–1^), tetracycline (2 μg mL^–1^), or enrofloxacin (0.25 μg mL^–1^), alone or in combination depending on selection needs. When needed, mycoplasmas were subcloned three times as previously described (Rosengarten et al., 1994), and the last broth culture was filtering through a 0.22 μm-pore filter before being stored at −80°C.

**TABLE 1:** Mating (M) and PEG-artificial fusion (H) experiments: Partners, chromosomal position of their respective selective markers (Tag), and name of transconjugants or PEG-hybrids.

**Partner 1**	**Tag position**	**Partner 2**	**Tag position**	**Transconjug. and Hybrids**	**Experimental conditions**		**References**
PG2G-181	648734	5632T-H3	529737	T1-1	Mating	M1	This study
				T1-2			
					
				H1-1	PEG-artificial fusion	H1	
				H1-2			
	
PG2G-71	473081	5632T-H3	529737	T2	Mating	M2	
	
PG2G-10	104815	5632T-H3	529737	T3	Mating	M3	
	
PG2T-3	770055	5632G-3	919899	T4-1	Mating	M4	
				T4-2			
				T4-3			
					
				H4-1	PEG-artificial fusion	H4	
				H4-2			
				H4-3			
				H4-4			

PG2E10	212329^∗^	5632G-3	919899	T5-1	Mating	M5	Faucher et al., 2019
	214190			T5-2			
	647997			T5-3			
				T5-4			
				T5-5			
				T6-1	Mating	M6	
					
				T6-4			
				T6-5			

*^∗^Position of chromosomal mutations conferring enrofloxacin resistance, all other antibiotic tags correspond to resistance gene randomly introduced by transposition.*

**TABLE 2:** Mating experiments using different *M. agalactiae* strains.

**Mating couples^∗^**				**Mating frequency**	**Progeny backbone (%)^#^**
		
**ICE**		**ICE**			
p5632^G^	+^§^	pPG2^T^	−	4.8 ± 1.1 × 10^–8^	100% 5632
p5632^G^	+^§^	p4867^T^	+	2.9 ± 1 × 10^–9^	70% 5632
					19% 4867
					11% 5632/4867¤
pPG2^G^	−	p4867^T^	+	1.2 ± 1.3 × 10^–9^	100% 4867
p5632^G^	+^§^	p14628^T^	+	1.1 ± 0.4 × 10^–9^	100% 5632
pPG2^G^	−	p14628^T^	+	0	N/A
p5632^G^	+^§^	p4055^T^	−	1 ± 0.03 × 10^–7^	100% 5632
pPG2^G^	−	p4055^T^	−	0	N/A

*^∗^Mating experiments were performed using, for each partner, a pool (p) of at least 10 tagged clones carrying the selective marker at different positions (see section “Materials and Methods”). ^§^ 5632 strain contains three essentially identical copies of ICE, other strains contain one copy. ^#^Percentage of the mating progeny having either one or the other parent backbone as defined using a specific PCR typing assay (see section “Materials and Methods”). ¤ Typing PCR results could not identify a dominant backbone. N/A, non-applicable.*

### Mycoplasma Genomic Tagging by Insertion of Stable Selective Antibiotic Makers

Antibiotic markers providing resistance to gentamicin (Gm) or tetracycline (Tet) were randomly introduced in the genome of Ma by using a modified version of the transposon Tn*4001* (mini-Tn) as previously described (Dordet Frisoni et al., 2013). Briefly, mycoplasmas were transformed with plasmid pMT85 which carries a mini-Tn composed of the gentamicin resistance *aacA–aphD* gene or the tetracycline resistance determinant *tetM* flanked by two Tn*4001* inverted repeats (IRs). Since the mini-Tn contains no transposase gene (it is located elsewhere in the pMT85), its insertion into the cell chromosome is stable (Baranowski et al., 2010). In addition to conferring antibiotic resistance to cell, this provides a proxy to trace chromosomal sequences that have been transferred. Insertion of the mini-Tn was monitored by PCR, and its chromosomal location determined by direct sequencing of genomic DNA as previously described (Dordet-Frisoni et al., 2014). An enrofloxacin-resistant (Enro^R^) strain of Ma PG2 (PG2^E10^) with mutated Enro target genes [three SNPs located in *parC* (214190), *parE* (212329), and *gyrA* (697997)] was obtained in a previous study (Faucher et al., 2019), after four passages in medium containing increasing concentrations of enrofloxacin (0.25, 0.5, 1, and 10 μg.mL^–1^). Mating with 4055, 4867, or 14628 strains with PG2 or 5632 were conducted using pools of 10 clones carrying the selective marker along the chromosome. This strategy was employed to enhance the chance of detecting recombination events between the different Ma genomes and avoid the negative effects of (i) the Tn insertion in a gene involved in conjugation and (ii) the colocalization of the markers in the donor and recipient genomes.

### Mating Experiments and Genetic Characterization of MCT Progenies

**FIGURE 1:**
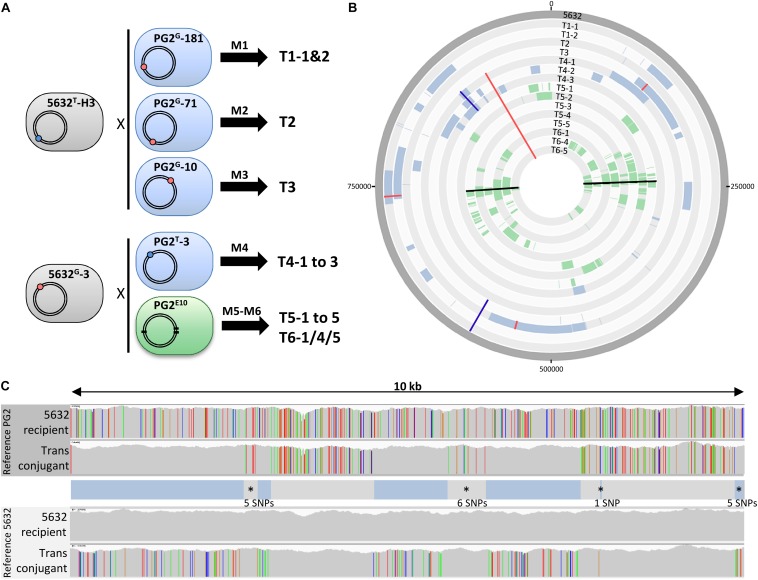
Mating experiments create a diversity of transconjugants with mosaic genomes. **(A)** Mating experiments (M1–M6) conducted to produce mycoplasma transconjugants, with M5 and M6 being two independent experiments that used the same parents. The 5632 and PG2 parent strains carried the Tet (blue dot) or Gm (red dot) genetic-marker located in different loci depending on the mating. In PG2^E10^ (M5 and M6), black thick lines correspond to the position of chromosomal mutations that confer enrofloxacin resistance (Enro^R^); **(B)** DNA plotter representation depicting the mosaic structure of the 15 Ma transconjugants. Sequences specific to PG2, PG2^E10^, and 5632 are color-coded in blue, green, and gray, respectively. The chromosome positions of the Gm-marker, the Tet-marker and the Enro^R^-mutations in transconjugants are indicated with red, blue, and black lines, respectively. From outer to inner circles: 5632, T1-1, T1-2, T2, T3, T4-1 to T4-3, T5-1 to T5-5, T6-1, T6-4, and T6-5. **(C)** Example of micro-complexity regions observed in mycoplasma transconjugants using T6-4. The 5632 recipient and the transconjugant T6-4 genomes were aligned to that of the PG2 (top) and 5632 (down) and the result obtained for a 10-kb segment is shown, as visualized with the IGV software. Each color bar represents a SNP with the four different colors representing the four different bases. The overall genotype of the T6-4 transconjugant is represented in between the alignments, with DNA segment corresponding to PG2 donor in blue and the 5632-recipient backbone in gray. Micro-complex regions are annotated by an asterisk. They correspond to small integrated PG2 fragments ≤10 SNPs, or PG2 acquired segments interrupted by a small region of 5632 recipient cell ≤10 SNPs. The number of SNPs in these micro-complex regions is indicated.

Mating experiments were conducted as previously described (Dordet Frisoni et al., 2013) using individual (Figure 1 and Table 1) or pools of marked clones mixed in equal proportions (Table 2). Briefly, donor and recipient cells were cultured for 24 h in SP4 medium and mixed in a final volume of 2 mL at a 1:1 volume ratio (about 10^9^ CFU mL^–1^). The mixture was centrifuged at 8000 rpm for 5 min and the pellet resuspended in 1 mL SP4 before further incubation at 37°C for 16 h. Cells were then seeded onto SP4-solid media supplemented with the appropriate antibiotics and incubated at 37°C for 3–4 days. Individual transconjugant colonies were randomly picked and grown into SP4 medium supplemented with gentamicin and tetracycline or gentamicin and enrofloxacin. Mycoplasma membrane cell fusion in the presence of PEG (polyethylene glycol 8000) was carried out as previously described (Teachman et al., 2002). The transconjugant frequency was determined as the number of transconjugants divided by the total CFU. Genomic DNA was extracted from mycoplasma cells using the classical phenol-chloroform method (Sambrook et al., 1989). PCRs were carried out for the detection of antibiotic resistance markers and for genomic backbone determination using oligonucleotides described in Dordet-Frisoni et al. (2014) for 5632 vs. PG2 transconjugants and others described in Supplementary Table S2. A maximum of 40 and a minimum of 12 transconjugants (for less efficient mating) were analyzed for each mating pair.

### Illumina Whole Genome Sequencing and Bioinformatic Analyses

DNA libraries and Illumina sequencing using HiSeq technology (paired-end, 2 × 150 bp, an average of 3000X for coverage depth) were performed according to the manufacturer’s instructions (Illumina, San Diego, CA, United States) at the GATC Biotech facility (Konstanz, Germany). Bioinformatics analyses were conducted using the GenoToul Bioinformatics facility, Toulouse, France^1^. Quality of the sequencing reads was controlled with FASTQC tool^2^. Each transconjugant or PEG-hybrid reads was respectively, aligned to the Ma genome PG2 55-5 (NC_009497.1), 5632 (NC_013948.1), and 4867 (SPQY00000000) using Burrows–Wheeler Aligner with MEM algorithm (Li and Durbin, 2009) with default parameters. The quality of the alignments was controlled with Qualimap 2.2.1 (mean coverage and mean mapping quality) (Okonechnikov et al., 2016) and SAMtools flagstat (Li et al., 2009). The resulting alignments were converted to BAM, indexed and sorted using SAMtools and visualized using the Integrative Genome Viewer (IGV 2.3.93) (Thorvaldsdóttir et al., 2013), Artemis 16.0.0 (Rutherford et al., 2000) and ACT 13.0.0 (Carver et al., 2005).

Detection of parental inherited fragments in transconjugants was possible because of the polymorphisms existing between the PG2 and 5632 genomes. As calculated by Breseq and Nucmer tools (Kurtz et al., 2004; Deatherage and Barrick, 2014), this polymorphism corresponded to 1 variation (SNPs or indels) every 26 nt between closely related nucleotide sequences. In addition, each nucleotide of genes present in one strain but not in the other was considered as a nucleotide variation, giving a final polymorphism of 1 variation every 11 nt. Alignment and motif searches in the region surrounding homologous regions were done with MEME/MAST software (V 5.0.1) (Bailey and Gribskov, 1998).

To define the genomic region of different parental origin with no ambiguity, reads mapping equally on both genomes were filtered out and mismatches were not allowed for reads specifically mapping to one of the two parental genomes, as previously described (see corresponding workflow in Faucher et al., 2019). Donor-specific reads, corresponding to inherited regions, were manually curated using Artemis and the Artemis BamView. For fragment delimitations, only reads with coverage higher than 15X and feature size of at least 25 nt were taken into account by selecting “Analyse” and “create features from coverage hit” in Artemis BamView. Fragments corresponding to (i) the *vpma* and *hsd* gene families that are subjected to frequent DNA rearrangements and contain several repeated sequences and to (ii) the duplicated *rrn* operon were manually curated and removed as well as fragments having no SNP based on Bam files. Circular plots were obtained with the Artemis DNA plotter tool. House-keeping genes and accessory genes were classified base on their E.C. number and the RAST (Rapid Annotation using Subsystem Technology) subsystems classification^3^. All gene that were not classified, and those classified in “Phages, Prophages, Transposable elements, Plasmids,” and “Virulence, Disease and Defense” were considered as accessory genes. *De novo* assembly of strain 4867 was done with Abyss v2.1.5 (Jackman et al., 2017) and resulted in 15 contigs and a total length of 923800 nt.

### Data Availability/Submission

All sequence data (fastq) are available at the EMBL database, the European Nucleotide Archive (ENA) at http://www.ebi.ac.uk/ena with the study numbers PRJEB27571 (accession number ERP109666). Two sets of transconjugant sequences were used in this study. The first set was selected from Faucher et al. (2019) and corresponds to transconjugants T5-1 to -5 (sample accession ERS2631597 to ERS2631601), transconjugants T6-1 (ERS2631602), T6-4 (ERS2631604), and T6-5 (ERS2631605). The second set was obtained during this study and corresponds to T1–T4 transconjugants (study numbers PRJEB27571, sample accession ERS2755466 to ERS2755472). From the same study, sequences of M161.11 and M171.4 transconjugants were available with the accession numbers ERS3559779 and ERS3559780, respectively. PEG-Hybrids (H1–H4) generated in this article have been deposited in a second study number PRJEB28807 (accession number ERP111063). The Whole Genome Shotgun project of 4867 strain used in this study has been deposited at DDBJ/ENA/GenBank under the accession SPQY00000000.

## Results

### MCT Creates Genome Mosaicism via the Co-transfer of Multiple, Unrelated Chromosomal Fragments

To decipher the features and extent of MCT, individual transconjugants were generated by mating using five different mycoplasma pairs (Figure 1A and Table 1) were analyzed. All pairs were derived from PG2 and 5632, two strains known as MCT-donor and -recipient, respectively, for which the circularized genome sequences are available. More specifically, two 5632-recipient clones, namely 5632^G^-3 and 5632^T^-H3, were chosen and have the gentamicin (Gm) or the tetracycline resistance (Tet) genes stably inserted at positions nt-919899 and nt-529737, respectively (Dordet-Frisoni et al., 2014). As donors, we selected (i) three PG2 clones having the Gm marker stably inserted at different positions (Table 1) (matings M1 to M3), (ii) the PG2^T^-3 clone having the Tet marker at position nt-770055 (mating M4), and (iii) the PG2^E10^ mutant which enrofloxacin resistant phenotype, Enro^R^, due to three point mutations located in two distinct opposite loci, in the *parE–parC* operon and the *gyrA* gene [matings M5 and M6 for which offsprings were already available from our previous work (Faucher et al., 2019; Figure 1A and Table 1)]. This experimental setting was implemented to address the influence on MCT (i) of the position and nature of the selectable marker and (ii) of applying the selective pressure on a single-locus (M1–M4) versus two distant loci (M5 and M6). Overall, a total of 15 individual transconjugants were selected from the five independent mating experiments (Figure 1A and Supplementary Table S1) and their genome were fully sequenced and analyzed.

Whole genome sequence (WGS) analyses of individual transconjugants first confirmed that all displayed a 5632 backbone in which chromosomal regions were replaced by their PG2 donor counterparts (Figure 1B). This exchange was evidenced by the concomitant loss of 5632 recipient-specific SNPs (Figure 1C), the detection of PG2 donor-specific SNPs and the acquisition of the PG2-selectable marker. In very rare occasions, sequencing reads matched both the donor and the recipient SNPs almost equally: their mapping corresponded to regions containing repetitive elements such as the *vpma* multi-gene family (Nouvel et al., 2009), for which precise organization and localization would require *de novo* assembly using long-read sequencing technology.

Comparative sequence analyses of the 15 individual transconjugants showed that during a single mating experiment, multiple distinct chromosomal regions of the recipient genome were replaced by donor sequences regardless of the mating pair tested. More specifically 3–30 fragments were exchanged, with 85% having no selectable marker (Figure 1B and Supplementary Table S3). These PG2 chromosomal segments were co-inherited without preferential bias except around the donor-selective marker where a higher proportion of donor DNA fragments were detected (see below). Overall, these transfer events resulted in a variety of mosaic genomes (Figure 1B), with no transconjugant displaying the same blend of parental genomes.

**FIGURE 2:**
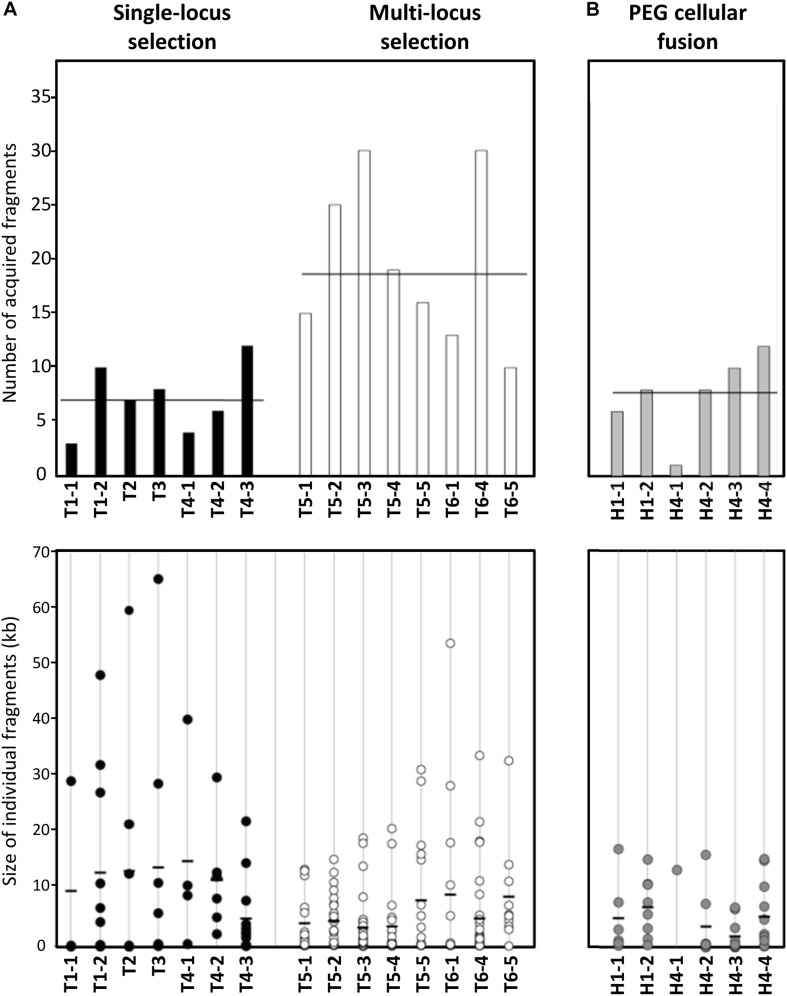
Number and size of PG2 donor fragments acquired by MCT in mating progeny. **(A)** Bar graph and dot plot chart illustrating the number and size of PG2 acquired fragments in all 5632 mating progenies (T1–T6 transconjugants). Data obtained with transconjugants produced by single-locus selection matings (see Figure 1 matings M1–M4) are represented by black-bar graph and -dots and those produced by multi-locus selection mating (see Figure 1 matings M5 and M6) by white-bar graph and -dots. The bar graph represents the number of PG2 donor fragments acquired by each transconjugant and average is indicated by horizontal thick line. The dot plot chart illustrated the size and the number of each PG2-fragment integrated into 5632 transconjugants. Each dot represents a donor-fragment, which is positioned in the graphic depending on its size (kbp). Black thick lines correspond to the mean size of all DNA fragments acquired in individual transconjugants. **(B)** Bar graph and dot plot chart representing the number and size of incorporated fragments in hybrids progeny obtained after PEG artificial-fusion (see Figure 3, H1-1, H1-2 and H4-1 to H4-4). As **(A)**, the bar graph represents the number of PG2 donor fragments and the dot charts the size and the number of each PG2 incorporated fragment in 5632 PEG-hybrids. The scale is the same as in **(A)**.

Sizes of the inherited PG2 fragments varied from 49 bp to 64.7 kb, with a 6.7 kb average and a mean of 14 chromosomal fragments acquired per genome (Figure 2 and Supplementary Table S3). Regarding the total amount of transferred DNA, mating progenies contained from 29.4 to 147.9 kb of PG2-donor sequences, representing 3.3–16.9% of the transconjugant genomes. The number of exchanged CDSs ranged from 20 to 163, with both house-keeping and accessory genes being almost equally affected by MCT (Table 3 and Supplementary Table S4). These findings supported our initial hypothesis that all PG2 chromosomal regions are potentially transferable.

**TABLE 3:** Genetic modifications induced by MCT in 5632-transconjugants (T) or -hybrids (H).

**Transconjugants**	**CDS loss (5632)**	**CDS gain (PG2)**	**Chimeric CDSs**	**Variations**	**SNPs**	**Indels**	**% of non-synonymous mutations**	**SNPs in intergenic regions**
**Single-locus selection matings**								
T1-1	1	2	4	630	580	50	22	81
T1-2	35^∗^	10	13	4092	3723	366	24	354
T2	13	29^#^	7	2759	2469	289	27	267
T3	7	8	12	3795	3453	341	21	247
T4-1	5	5	5	1839	1702	136	25	111
T4-2	28^∗^	5	9	2415	2230	185	25	230
T4-3	9	7	15	2007	1811	194	29	174
**Multi-locus seletion matings**								
T5-1	2	4	20	2353	2190	163	21	158
T5-2	26^∗^	7	23	3584	3349	235	22	251
T5-3	7	12	35	2625	2410	213	22	240
T5-4	4	3	17	2067	1916	151	20	214
T5-5	28^∗^	10	15	3871	3511	356	25	356
T6-1	23^∗^	7	11	3508	3280	227	23	333
T6-4	9	9	28	4028	3742	284	21	336
T6-5	2	11	16	2706	2500	205	23	266
**PEG artificial-fusion of mycoplasma cells**								
H1-1	0	2	6	972	881	91	24	114
H1-2	4	3	13	1669	1517	150	19	151
H4-1	4	3	2	17	10	7	50	2
H4-2	4	4	7	873	783	90	32	72
H4-3	0	0	0	734	688	46	21	31
H4-4	5	4	14	2086	1936	150	22	143

*^∗^loss of an ICE. ^#^Gain of a vestigial ICE from the PG2 chromosome.*

Despite the high synteny existing between the 5632 and PG2 genomes, some transconjugants gained up to 29 CDSs (3% of PG2 donor genome) or lost up to 35 CDSs (3.8% of the 5632 recipient genome) (Supplementary Table S4). In five transconjugants, gene losses were mainly due to DNA recombination occurring in chromosomal regions adjacent to ICEA, an integrative conjugative element of 23 kb that is present as three almost identical ICEA copies in the 5632 recipient strain. These events resulted in ICEA deletion (ICEA-III in T5-5 and ICEA-I in T1-2, T4-2, T5-2, and T6-1) and sequence analyses confirmed that this was not due to ICEA self-excision (Dordet-Frisoni et al., 2013), but to DNA replacement with PG2 syntenic regions having no ICE, as CDSs were exchanged from both sides of the ICEA deletions (7 CDSs for T1-2, 83 CDSs for transconjugant T4-2, 6 CDSs for T5-2, 28 CDSs for T5-5, and 2 CDSs for T6-1) (Supplementary Table S4). In another transconjugant, T2, the PG2 fragment included a PG2-ICEA vestige having no counterpart in 5632, permitting the gain of 26 new CDSs, the vast majority of which were annotated as pseudogenes (Supplementary Table S4). Thus loss or gain of regions containing ICEA following MCT had a direct impact on the overall gene content and genome size considering the extremely small mycoplasma gene content (about 800 CDSs for Ma genomes).

Gain or loss also affected CDSs that belong to the accessory genome and were not ICEA-encoded. A high proportion of these was annotated as hypothetical protein (71% of acquired CDSs and 50% of lost CDSs) and over 10% of the exchanged CDSs corresponded to membrane lipoproteins (Supplementary Table S4). When compared to the PG2 donor, strain 5632 genome possess four additional genes that encode two type II restriction modification (RM) systems (MAGa3950 and MAGa3970; MAGa4250, and MAGa4260) and one CDS coding for a type-III methylase (MAGa1570) (Supplementary Table S4) (Nouvel et al., 2010). During MCT, replacements can result in these being deleted as illustrated by transconjugant T2 which has lost all four CDSs (MAGa3950-3970, MAGa 4250-4260) belonging to the two specific 5632 type-II RM systems (Supplementary Table S4).

Mycoplasma chromosomal transfer is a powerful process of horizontal, distributive chromosomal transfers that generates large scale genomic changes, with the replacement of complete genes as well as the gain or loss of large blocks of genetic material.

### MCT Introduces Micro-Heterogeneity in Mycoplasma Mosaic Genomes

Comparative genome analyses at the nucleotide level revealed that the total number of SNPs introduced by MCT in the recipient 5632 chromosome varied from 630 to 4092 nt, with a mean of 2590 base substitutions and 226 indels (Table 3). Overall, 90% of these variations were located within CDSs as expected from a genome with gene density of 88.5 ± 0.5%.

Most PG2 aquired regions corresponded to several kb DNA fragments, containing an average of one variation per 11 nt (see section “Materials and Methods”). Our analyses also concomitantly uncovered micro-complex regions (Figure 1C) that were composed (i) by a few donor SNPs (arbitrarily, regions containing ≤10 of donor SNPs) or (ii) by inherited PG2 fragments interspaced by short intervals (≤10 SNPs) of the 5632-recipient DNA (Figure 1C and Supplementary Table S5). These micro-complex regions represented between 8 to 35% of the transferred materials (Supplementary Table S5) and were not *de novo* mutations as they were clearly of parental inheritance, as illustrated in Figure 1C. Most likely, these micro-complex regions were the result of a combination of “recombination and repair events” occurring between the recipient chromosome and one, or more, of the transferred donor DNA fragments. Interestingly, about 25% of the SNPs introduced by the donor in these micro-complex regions (≤10 SNPs) caused an amino acid substitution when compared to the original 5632 parental products (data not shown). This proportion increased in smaller micro-complex regions (≤5 SNPs) in which more than 40% of the SNPs caused non-synonymous mutations (Supplementary Table S6).

Another source of micro-complexity was provided by chimeric CDSs, a combination of both parental sequences most likely resulting from one or more recombination events within CDSs themselves. These chimeric CDSs were found in all transconjugants, with their number varying from 4 to 35 (Table 3 and Supplementary Table S4), and corresponded to housekeeping, as well as accessory genes, introducing numerous SNPs in gene sequences, such as in *parC* (45 SNPs in T3, 97 SNPs in T5-2), *ruvB* (9 SNPs in T4-3), *recA* (19 SNPs in T5-3 and 9 SNPs in T5-4), or in the P30 lipoprotein gene (40 SNPs in T5-1).

### The Type of Selection Pressure Affects the Level of Complexity of Transconjugant Mosaic Genomes

Comparison of transconjugant genomes derived from the same parental pair (T1-1 to -2, T4-1 to -3, T5-1 to -5, or T6-1/4/5), revealed that the number of exchanged chromosomal fragments varied on average by 3-fold and the percentage of transferred PG2 genome by 2.2-fold among siblings (Figure 2 and Supplementary Table S3). The position and the nature of the selective marker, Gm versus Tet, did not influence the extent of genome mosaicism: the number of exchanged DNA fragments was comparable between progenies generated by 5632^T^/PG2^G^ pair (mating M1, M2, and M3 depending on the Gm position in the donor chromosome) or by the 5632^G^/PG2^T^ pair (mating M4) with a mean of 7 fragments (*p* = 0.9, Student’s *t*-test). Of note, the percentage of transferred PG2-DNA was slightly higher (10.4%) with the 5632^T^/PG2^G^ pair than with the 5632^G^/PG2^T^ (7.3%) (Supplementary Table S3). This may be due to the respective position of the recipient and donor markers: the closer they get in the final transconjugant progenies, the more the risk of losing the donor marker increased during incorporation of the donor fragment carrying the selective marker (Figure 1).

When more than one donor chromosomal region was required for adaptation (multi-locus selection pressure), here when using PG2 Enro^R^ mutant (PG2^E10^) as donor (matings M5 and M6), we observed a higher degree of mosaicism of the genome progenies (Figures 1, 2). Indeed, T5 and T6 transconjugants had to acquire two diametrically opposed chromosomal mutated regions: *parE-C* (212329–214190) and *gyrA* (647997) to withstand the enrofloxacin selective pressure (Faucher et al., 2019). T5 and T6 Enro^R^ transconjugants, in fact, did inherit a mean of 19.8 ± 7.7 donor fragments compared to 7.1 ± 3.2 when the selection pressure applied on a single locus of the donor (mating M1–4) (Supplementary Table S3). Interestingly, in both cases, the total amount of acquired PG2-DNA was comparable (11.8% ± 3.4% for T5-6 Gm/Enro^R^ transconjugants versus 9.1% ± 3.9 for Gm/Tet (*p* = 0.36, Student’s *t*-test). Compared to single-locus selection matings (M1 to M4), the acquired fragments in progenies selected under multi-locus selection were smaller with an average of 11.7 kb versus 5.8 kb, respectively (Figure 2 and Supplementary Table S3). The mosaicism surrounding the selectable regions was higher in T5- and T6-Enro^R^ progenies, with 40% of the exchanged fragments mapping within 50 kb from the donor Enro^R^ mutations compared to 20% for progenies derived from a donor having the selectable marker, Gm or Tet, inserted at one locus (Figure 1B). Furthermore, the percentage of micro-variations related to the remnant 5632-DNA present between the PG2-donor regions was twice as high in T5 and T6 progenies than in “single-locus” selection matings (Supplementary Table S5). T5 and T6 progenies, which selection relied on the acquisition of diametrically opposed chromosomal regions, have apparently undergone a high rate of recombination events especially in regions surrounding the chromosomal selectable mutations. Yet, the overall amount of integrated PG2-donor DNA was similar to that observed in mating involving a single-locus selection (Supplementary Table S3).

### PEG Artificial Fusion of Mycoplasma Cells Generates Genome Mosaics of Lower Complexity

**FIGURE 3:**
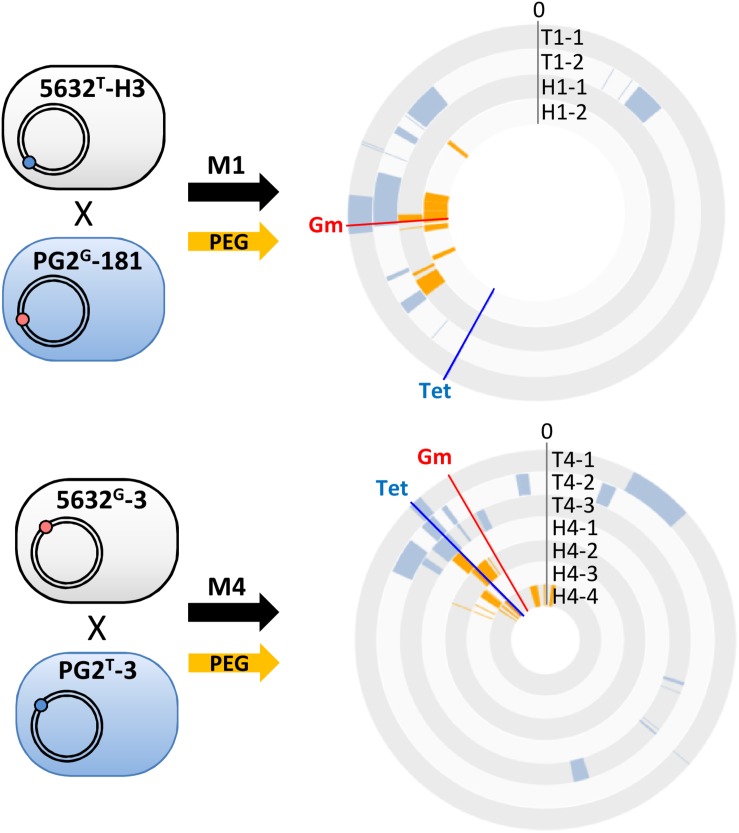
Polyethylene glycol artificial-fusion of mycoplasma cells also results in mosaic genomes. PEG artificial-fusions were conducted with the same pairs as in mating M1 and M4 and resulted in PEG-hybrids (H1-1, H1-2 and H4-1 to H4-4). 5632 and PG2 parents carried the Tet (blue dot) or Gm (red dot) genetic-marker at different loci. The DNA plotter depicts the mosaic nature of the five transconjugants (T1-1, T1-2 and T4-1 to T4-3) and the 6 PEG-hybrids (H1-1, H1-2 and H4-1 to H4-4). PG2-specific sequences are color-coded in blue for transconjugants, orange for PEG-hybrids, and the 5632 genome backbone is depicted in gray. Chromosomal positions of Gm- and Tet-markers in progenies are indicated with red and blue lines, respectively. Outer circles: 5632, T1-1 and T1-2, H1-1, H1-2 and inner circles T4-1 to T4-3, H4-1 to H4-4.

In *M. agalactiae*, HGT occurs only when at least one mating partner carry a functional ICE (Dordet-Frisoni et al., 2013; Baranowski et al., 2018), but we have previously demonstrated that mycoplasma conjugation could be artificially bypassed by using PEG, a polymer which induced membrane fusion, resulting in chromosomal replacement independently from ICE-encoded conjugative properties (Dordet-Frisoni et al., 2014). To gain further insights into parameters affecting genome mosaicism, parental pairs previously used in M1 and M4 matings (Figure 1A), were respectively, fused using PEG as previously described (Dordet-Frisoni et al., 2014) and six individual, double-resistant progenies, H1-1 and -2 and H4-1 to -4 were selected (Table 1). Their genomes were analyzed as above (Figure 1 and Supplementary Table S3) and their overall structure was compared to transconjugants (T1 and T4) derived from the same pairs following classical mating conditions (Figure 3).

As for classical conjugation, all PEG-hybrid progenies possessed a 5632 backbone, with several incorporated PG2-DNA fragments and no influence of the nature and position of selection marker. The number of PG2-recombined fragments was similar to that observed during “single-locus” selection matings with a mean of 7.5 ± 3 exchanged DNA regions (Figure 2 and Supplementary Table S3). As for multi-locus selection matings, the mean size of PG2 incorporated-DNA segments was smaller than for “single-locus” selection (11.7 kb) with an average of 6.1 kb (Figure 2 and Supplementary Table S3). PEG-hybrid genomes showed less complexity compared to T1 and T4 transconjugants, with the number of gained and/or lost CDS, as well as the number of acquired SNPs, being lower (Table 3). In H1 and H4 genomes, PG2 incorporated fragments were mostly concentrated around the selection marker with 45.5% of the exchanged fragments that mapped within 50 kb from the selection marker compared to 18.6% for M1 and M4 progenies (Figure 3). Despite a greater simplicity of the PEG-hybrid genomes, this result suggests that the recombination process observed for MCT does not require the presence of the conjugative pore machinery expressed by ICE during conjugation. Analysis of the PEG-hybrid genomes reinforces the hypothesis that the ICE mainly contributes to promote cell-to-cell contact, bridging the cell cytoplasmic content so that chromosomal exchange may take place. These data also suggested that specific properties of the recipient or donor cells are responsible for the apparent directionality of the transfer rather than the transfer preferentially occurring in a given direction, as further discussed.

### Chimeric Genes and Micro-Complex Regions Reflect a Highly Permissive Recombination System

**FIGURE 4:**
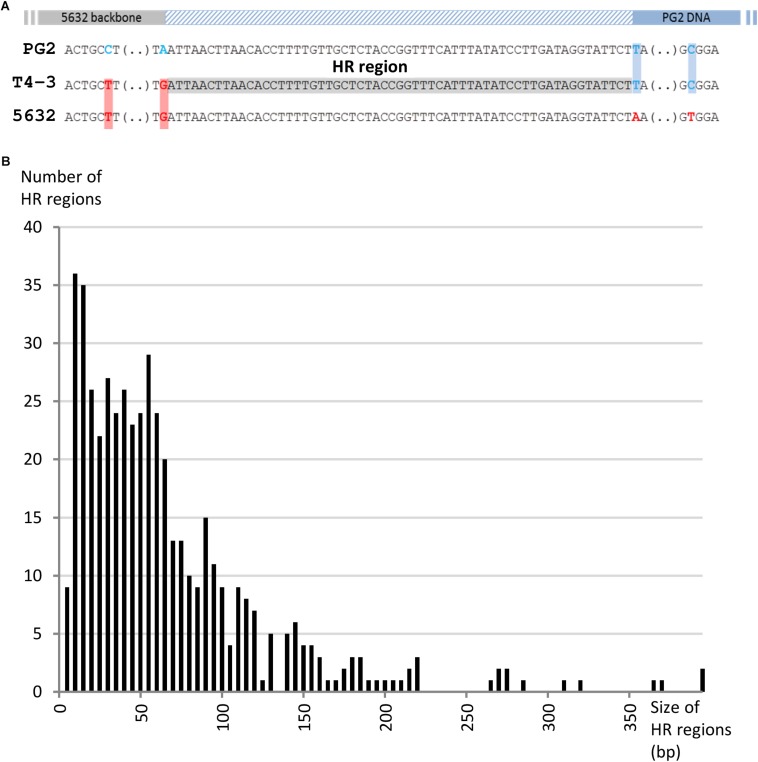
Analyses of homologous region (HR) flanking the acquired DNA in transconjugants. **(A)** Homologous regions (HR) were defined as identical sequences in between donor (gray) and recipient DNA (blue) that were delimited at one end by the first recipient-SNP (boxed in red) and, at the other, by the first donor SNP (boxed in blue) encountered on the chromosome. Only homologous regions with coverage of at least 15X were taken into account and are represented here by a hatched blue rectangle. **(B)** Bar graph illustrating the number of HR depending on their size (bp).

When MCT was first discovered, we assumed that the replacement of chromosomal recipient-loci by their donor-counterparts occurred via homologous recombination. Here, Illumina sequencing of 21 individual mosaic genomes provided a unique opportunity to revisit this issue. We then focused on homologous regions (HRs) flanking the inherited donor-fragments of both the transconjugants and PEG-hybrid genomes. HRs were defined as identical sequences in between donor and recipient DNA that were delimited at one end by the first recipient-SNP and, at the other, by the first donor-SNP encountered on the chromosome (Figure 4A). Alignment and analyses of HRs surrounding sequences did not reveal any specific motif, structure or bias in GC content. HRs length varied from 2 to 394 bp, with a mean of 64 bp, and no difference in HRs length was observed between the different experiments (Figure 4B and Supplementary Table S7).

The Minimal Efficient Processing Segment (MEPS), previously defined by Shen and Huang (1986) as the shortest length of sequence homology necessary for efficient recombination, was shown to significantly vary among organisms (Shen and Huang, 1986; Kung et al., 2013). In *E. coli*, efficient recombination has been observed with as little as 23 bp of sequence homology. For mycoplasmas, MEPS has never been determined, but here, data showed that in Ma transconjugant genomes 21.5% of HRs were micro-homologous regions of less than 20 bp (Figure 4B), 27% of which were present on both sides of the exchanged fragments. The proportion of small HRs is the same regardless of the type of offspring analyzed (24 ± 2.7% of HRs < 20 nt for single-, multi-locus selection transconjugants and PEG-hybrids). This high proportion of micro-HRs raised the question of the existence of an illegitimate (non-homologous) recombination (IR) mechanism that would join DNA molecules at sites with only a few identical nucleotides.

### Features of MCT Recipient Versus Donor Cells

So far, chromosomal exchanges were only documented between strains PG2 and 5632 of Ma as well as in between the two related species Ma, strain 5632, and *Mycoplasma bovis* strain PG45 (Dordet-Frisoni et al., 2014). Each time, the detection of transconjugants required the presence of the 5632 strain that carries three, functional copies of ICEA; each time their fine genetic analyses designated 5632 as the recipient strain (Dordet-Frisoni et al., 2013; Baranowski et al., 2018; Faucher et al., 2019). This puzzling observation prompted us to address the conjugative properties of three other Ma strains, namely 4867, 4055, and 14628 that differ in their ICE content and life history (e.g., year of isolation, geographical origin, ICE number) (Supplementary Table S1). For this purpose, each of these strains was mated with 5632 or PG2.

To maximize the chance of selecting transconjugants, matings were performed with a pool of clones carrying the Tet-selective marker at different loci of the strains to be tested and with a pool of Gm-tagged PG2 or 5632 (Table 2). Most mating experiments yielded double-resistant mycoplasmas with frequencies ranging from 1.1 × 10^–9^ to 1 × 10^–7^ transconjugants/total CFU. As expected from previous data, the mating couple involving the two ICE-negative strains PG2 and 4055 gave no transconjugant, the presence of an ICE in at least one mating partner being required for conjugation to occur (Dordet-Frisoni et al., 2013). A negative result was also repeatedly obtained when using the mating couple PG2-14628 (Table 2). Since previous data suggested the occurrence of a functional ICE in 14628 (Tardy et al., 2015), this result was unexpected and raised the prospect that other cell factors might be needed for MCT to succeed or that there is a barrier to HGT in mating with this recipient strain.

All transconjugants were shown to display the 5632 backbone except for mating involving strain 4867 (Table 2). With this particular strain, all progenies generated with the PG2 as mating partner displayed a 4867 genomic backbone while both parental backbones were detected in progenies obtained with 5632 (Table 2). The genomes of strain 4867 and of two transconjugants with a 4867 backbone, namely M161.11 from 5632 × 4867 mating and M171.4 from PG2 × 4867 mating, were sequenced by Illumina. WGS analyzes indicated that 4867 is a simplified version of the 5632 strain as both genomes differ by 1084 nucleotide variations (Supplementary Table S8). Strain 4867 contains only one ICE copy instead of three in 5632, and lacks the IS*30*-like transposase present in multiple copies in the 5632 genome (Supplementary Table S8). These differences enable the identification of the transconjugant genomic backbones but do not allow precise mapping of the chromosomal DNA exchanges in M161.11 transconjugant. The PG2 × 4867 transconjugant M171.4, however, contained six PG2-inherited DNA fragments, with two being large distant chromosomal DNA region of 23 and 42 kb (Supplementary Figure S1), while the remaining corresponded to micro-complex regions containing from 2 to 18 SNPs corresponding to the PG2-parental strain (Supplementary Figure S1).

Overall, these data showed that not only 5632 but also 4867, a strain phylogenetically very close to 5632, can act as a recipient strain in MCT. Since 4867 contains only one ICEA, mating data obtained here with the ICE-negative PG2 strain demonstrated that only one copy of this element is needed for MCT to occur. The genomic simplicity of strain 4867, when compared to 5632, makes it a good candidate to further decipher the factors involve in MCT.

## Discussion

This study formally supports our recent work and established MCT as a powerful conjugative process for extensive diversity within the *M. agalactiae* species. The limitless number of different mosaic genomes that may be produced by this phenomenon challenges our view on the biology of the simple mycoplasma cell and may have a tremendous biological impact in pathogenesis and adaptation to changing environments. More specifically, this study demonstrated that the horizontal, co-transfer of multiple, unrelated chromosomal regions is the rule rather the exception and that its extent is modulated by the use of certain antibiotics.

As shown by combining experimental mating experiments with WGS analyses of clonal progeny, MCT allows a recipient genome to simultaneous acquire, multiple, large or small donor DNA fragments. In a single mating experiment, two clonal populations can generate a diversity of unique blends of chimeric/mosaic progeny. MCT is an unusual conjugation process in that virtually any loci can be transferred in any combination as long as the resulting mosaic genome is viable. A similar unconventional conjugative mechanism termed distributive conjugal transfer (DCT) has been described in *M. smegmatis* by Gray et al. (2013). Both systems generate extensive genome mosaicism and, to some extent, resemble eukaryote meiosis: In mycobacterial DCT or in MCT, 20–25% of donor DNA can be exchanged with their recipient orthologues and distributed around the chromosome into up to 30 large and small fragments (Derbyshire and Gray, 2014). While MCT and DCT outcomes may appear identical from a genomic point of view, these systems belong to two distinct groups of bacteria that have little in common: mycoplasma and mycobacteria are phylogenetically remote, differ in their cell-envelope, and have respectively, small and large genomes with low and high GC content. As for conjugation, one main difference between DCT and MCT resides in their secretory systems. In mycobacteria, two Type VII secretion apparatus, ESX1- and -4 are essential for DCT and are, to our knowledge chromosomally encoded (Gray and Derbyshire, 2018). In contrast, no Type VII secretion system has been detected in mycoplasma, but several species possess a simplified version of Type IV Secretion System (T4SS) which is carried by an Integrative and Conjugative Element (ICE) (Calcutt et al., 2002; Marenda et al., 2006; Dordet-Frisoni et al., 2013; Baranowski et al., 2018).

Our group has previously demonstrated that such ICE elements are self-transmissible and play a key role in mycoplasma conjugation (Dordet-Frisoni et al., 2013). Hence, for MCT to occur, at least one of the two partners should carry an ICE (Dordet-Frisoni et al., 2013, 2014) and recent functional analysis confirmed the essentiality of ICE-encoded factors in conjugation (Baranowski et al., 2018). Mycoplasma conjugation is a complex phenomenon as two concomitant events take place during mating: the horizontal ICE self-transmission and chromosomal transfer, both occurring with the same frequency (Dordet-Frisoni et al., 2014). Indeed, ICE can disseminate from ICE-positive to ICE-negative cells while the chromosomal transfer is observed in the opposite direction, from ICE-negative to ICE-positive cells, both DNA movements being physically independent (Dordet-Frisoni et al., 2014). This and data obtained with mating experiments involving different *M. agalactiae* strains, with or without ICE, suggest that the presence of a T4SS is only required in the recipient cell. For mycobacterial DCT, ESX secretion apparatuses have been demonstrated to be essential both for donor and recipient cells (Gray and Derbyshire, 2018), with no mobile genetic elements or ICE being involved.

Wall-less mycoplasma cells can be artificially fused using PEG to bypass the first steps of conjugation (Dordet-Frisoni et al., 2014). This provides a wonderful means to address the influence of conjugative properties on DNA exchange and recombination. To our surprise, analyses of hybrid genomes generated by PEG artificial-fusion also resulted in mosaic genomes all having the genetic background of the “ICE-positive strain.” Thus, the apparent polarity of MCT (see above) might not be due to the ICE-conjugative machinery but rather to cytoplasmic factors that allow, or not, the survival and/or the incorporation of foreign DNA. Indeed, a difference in genes encoding DNA restriction modification systems exists between the partners used in this study (Nouvel et al., 2010), with the ICE itself encoding a hypothetical DNA methyl-transferase (Marenda et al., 2006; Baranowski et al., 2018). The role of these factors in MCT remains to be addressed. Interestingly, hybrid genomes obtained following PEG artificial-fusion were less heterogeneous than those obtained following conjugation, with the exchanged fragments being more concentrated around the selective marker (Figure 3). Identifying the factors responsible for this observation might not be trivial but will contribute to our understanding of the dynamics of the recombination events during MCT. In particular, one key question is whether multiple recombination events occur at the same time or sequentially, with their number depending on the duration of cell-to-cell contact.

When MCT was first discovered, our initial hypothesis was that DNA exchanges occurred via homologous recombination (Dordet-Frisoni et al., 2014). This was based on the analyses of a limited number of single transconjugant genomes and data further gathered in the current study indicate that this hypothesis might not always be valid. For homologous recombination to occur in classical bacteria, RecA must recognize a sequence of sufficient similarity during strand exchange which corresponds to the minimal efficient processing segment (MEPS) (Shen and Huang, 1986). MEPS has never been determined for mycoplasmas but in *E. coli* its length depends on the recombination pathway: it is between 23 and 27 bp for the RecBC pathway and between 44 and 90 bp for RecF-dependent pathway (Shen and Huang, 1986). Homologous Regions (HR) flanking donor DNA in mycoplasma transconjugant genomes were relatively short, with almost a third being less than 30 nt and ca. 7% ≤ 5 nt. Since mycoplasma genomes lack the MutSHL recombination system, these organisms might be more tolerant for sequence dissimilarity than *E. coli* or other classical bacteria (Sirand-Pugnet et al., 2007) and short HRs may reflect the high tolerance of the recombination machinery for dissimilarities as a consequence of an imperfect mismatch repair system. Mosaic genomes generated during MCT or artificial cell fusions are thus most likely the results of both homologous and illegitimate recombinations. The latter joins DNA molecules at sites of few or no homology in certain bacteria (Brigulla and Wackernagel, 2010). During natural transformation, some bacteria (de Vries and Wackernagel, 2002; Prudhomme et al., 2002; Meier and Wackernagel, 2003) used a homology-facilitated illegitimate recombination (HFIR) mechanism which enables the integration of foreign DNA (Brigulla and Wackernagel, 2010). HFIR involved an illegitimate recombination event in the foreign DNA adjacent to a homologous DNA (the anchor region) and occurred mostly within short stretches of sequence identity (3–12 nucleotides) with increased GC content. In our study, micro-HRs have a mean GC content of 29.1 ± 1.6%, a value not significantly different from the 29.7% calculated for the whole genome. This suggested that a system different than HFIR may occur in mycoplasma. In *E. coli*, two short-homology-independent illegitimate recombination systems have been described that are either mediated by DNA gyrase (Shimizu et al., 1997) or topoisomerase I (Bierne et al., 1997).

Interestingly, WGS analyses of Enro^R^ transconjugants with mutations in *gyrA* revealed that their mosaic genomes are more complex than in other matings. One difference was the need for these transconjugants to acquire two diametrically opposed chromosomal mutated regions to withstand the enrofloxacine selection pressure (Faucher et al., 2019). This constraint may have induced and/or promoted the selection of highly recombinogenic transconjugants. Another explanation is the use of fluoroquinolone which inhibits the bacterial DNA gyrase. Mutations in *gyrA*, and thus changes in the primary sequence of subunit A, as well as inhibition of the DNA gyrase by the quinolone have been shown to enhance illegitimate recombination and confer a hyper-recombination phenotype in *E. coli* (Ikeda et al., 1981; Ashizawa et al., 1999). These data raised the question of a similar role of the DNA gyrase in the genomic complexity of MCT offspring. This was further supported by the analyses of three Enro^S^ transconjugants (Faucher et al., 2019) (T6-3, T6-7, and T6-10 of mating M6, Supplementary Figure S2) which displayed a high degree of genomic mosaicism (mean of 14 fragments of 5.5 kb) and the mutated-*gyrA* of the PG2E10 donor but not the mutated *parE*–*parC* or *gyrB* loci. Additional data are needed to show whether *gyrA* mutations and/or the addition of enrofloxacin enhance mycoplasma genome mosaicism during mating.

Mycoplasma chromosomal transfer generates a unique blend of the parental genomes by combining macro genetic exchanges involving entire CDSs, operon and loci, with micro HGT-events which introduced micro-variations at the nucleotide level. A previous comparative genomic analysis suggested that 134 CDSs of the Ma genome had undergone HGT with members of the Mycoides cluster (Sirand-Pugnet et al., 2007). These transferred CDSs were distributed around the genome as clusters, an organization we thought reflected successive past HGT events. Experimental data gathered here and elsewhere support our initial *in silico* findings, and further demonstrated that MCT is both a past and contemporary phenomenon that has shaped mycoplasma species (Citti et al., 2018). Comparative sequence analyses suggested that the *Mycoplasma leachii* genome is a mosaic of the *Mycoplasma capricolum* and *Mycoplasma mycoides* genomes, both species belonging to the Mycoides cluster (Tardy et al., 2009). Indeed, this cluster is composed of species infecting ruminants, many of which are pathogenic and share the same habitat as *M. agalactiae*. Since most are known to harbor ICE elements (Tardy et al., 2015), these ruminant mycoplasmas may have retained the ability to conjugate and promote HGT among and within species.

An increasing number of mycoplasma genomic studies are detecting past HGT events (Citti et al., 2018; Lo et al., 2018), most often in species sharing the same host and in which ICE elements were identified. For instance, five gene clusters were predicted to have undergone HGT in between the phylogenetically distinct species *Mycoplasma hominis* and *Ureaplasma parvum* (*Mycoplasmataceae* family), two colonizers of the human urogenital tract (Pereyre et al., 2009). These data suggest that MCT is not restricted to *M. agalactiae* or to ruminant mycoplasmas.

During MCT and depending on the genome synteny, CDSs can be gained or lost, regardless of whether they are housekeeping or accessory genes. These events can result in the acquisition or the loss of entire biological pathways or of key adaptive properties and may have major impacts for mycoplasma survival and host-interactions. For instance, *M. agalactiae* has inherited an oligopeptide transport system from a Mycoides cluster ancestor which participates in a range of biological events, including: biofilm formation, antimicrobial-compound production, and adaptation to specific environments such as milk from which the pathogen is often isolated (Sirand-Pugnet et al., 2007). In the current study and in *in silico* analyses (Sirand-Pugnet et al., 2007), a large number of exchanges included genes encoding surface lipoprotein and membrane protein were observed. In the absence of a cell wall, the mycoplasma membrane constitutes the primary interface with the host environment. Thus, change, acquisition or loss by MCT of this category of genes can dramatically affect the interaction with the host. For instance, mycoplasmas having acquired the MIB-MIP gene tandem via HGT may have an advantage in withstanding the humoral host response because of their products inducing the capture and cleavage of IgG (Arfi et al., 2016). MCT may also have a tremendous impact on diagnostic, typing, or epidemiological studies by blurring the border in between species and strains. Hence, extensive HGT in human ureaplasmas has questioned the utility of serotyping for diagnostic purposes (Xiao et al., 2011).

Simultaneously to the exchanges of large fragments, micro-complex regions are created, inducing minor nucleotides substitutions and sequence alteration. Yet, these fine modifications may be responsible for important phenotypic changes, such as the acquisition of a high-level of antibiotic resistance following the transfer of a few point mutations (Faucher et al., 2019).

Overall, the mycoplasma minimal cells have retained a form of bacterial sex outcomes that may have a tremendous impact on their evolution and adaptability to complex hosts. The chromosomal transfer described here for mycoplasmas, by Gray et al. (2013) for Mycobacteria (Gray and Derbyshire, 2018), and by an increasing number of reports for other bacterial genera (Budroni et al., 2011; Lesic et al., 2012) most likely operates via different conjugative mechanisms but the end-process is identical, indicating that convergent evolution works toward creating ever-increasing diversity. Currently, the occurrence and role of DCT in bacteria are probably underestimated due to the difficulties in following these events within genetically closely related strains or species (Mortimer and Pepperell, 2014) and in distinguishing DCT from other HGT events related to mobile genetic elements or to natural transformation. Thanks to their small-size genome, mycoplasmas offer a simplified model (i) to decipher the mechanism of DNA transfer and integration responsible for this extraordinary plasticity, and (ii) to address the biological impact of such process, in particular for important pathogens.

## Data Availability Statement

The datasets generated for this study can be accessed from the European Nucleotide Archive (ENA) accession number ERP109666 contains two set of transconjugant: The first set was selected from Faucher et al. (2019) samples accession: ERS2631597 to ERS2631601, ERS2631602, ERS2631604, and ERS2631605. The second set was obtained for this study PRJEB27571, sample accession: ERS2755466 to ERS2755472, ERS3559779, and ERS3559780. For PEG-Hybrids the study number is PRJEB28807, accession number ERP111063.

## Author Contributions

ED-F, LN, FT, and CC designed the experiments. ED-F, MF, and ES performed the experiments. ED-F, MF, LN, CC, and EB analyzed the data. ED-F and CC drafted the manuscript.

## Conflict of Interest

The authors declare that the research was conducted in the absence of any commercial or financial relationships that could be construed as a potential conflict of interest.

## Footnotes

1 http://bioinfo.genotoul.fr/

2 http://www.bioinformatics.babraham.ac.uk/projects/fastqc/

3 http://rast.nmpdr.org/

## Supplementary Material

FIGURE S1 Mosaic genome of the M171.4 transconjugant derived from PG2 × 4867 mating. Position and length of PG2 donor fragments present in the M171.4 transconjugant having a 4867 genomic backbone. The DNA plotter depicts the mosaic nature of the M171.4 transconjugant. Chromosomal positions of Gm- (nt-922013) and Tet-markers (nt-20014) are indicated with red and blue line respectively. PG2-specific sequences are color-coded in blue, the 4867 and PG2 genome backbones are depicted in yellow and blue, respectively. From outer to inner circles 4867, M171.4, and PG2.

FIGURE S2 Features of EnroS transconjugants having acquired a donor mutated-*gyrA* but no *parE/parC* donor. **(A)** DNA plotter presenting the reconstructed mosaic genomes of transconjugants T6-3, T6-7, and T6-10 that derived from mating M6. These displayed a MIC value of 0.5 μg.mL^–1^ and were classified as Enro^S^. Their 5632 genomic backbones are depicted in gray and PG2 specific donor-sequences in green. Chromosomal positions of the 5632-Gm marker (nt-922013) and the acquired PG2 donor *gyrA* mutation (nt-647997) are indicated by a red and a black line respectively. From outer to inner circles 5632, T6-3, T6-7, and T6-10. **(B)** Dot plot chart illustrating the number and size of each PG2 fragments incorporated in transconjugants obtained after M1–M6 matings (see Figure 1). Each dot represents a donor-fragment positioned on the graphic depending of its size (kbp). Black thick lines correspond to the mean size of all DNA fragments acquired in individual transconjugants. **(C)** Bar graph representing the number of PG2 donor fragments acquired by each transconjugants. The average number of acquired DNA fragments is represented by a line. For both graph, **(B,C)**, data obtained with transconjugants produced by single-locus selection matings (see Figure 1; matings M1–M4) are represented by black-dots or bars and those produced by multi-locus selection matings (see Figure 1; matings M5 and M6) by white dots or bars. Transconjugants T6-3, T6-7, and T6-10 are depicted by green dots or bars.

TABLE S1 *Mycoplasma agalactiae* strain features.

TABLE S2 Oligonucleotides used in this study.

TABLE S3 Extent and size of PG2 donor chromosomal DNA fragments acquired during mating and PEG artificial-fusion.

TABLE S4 PG2-CDSs acquired in transconjugants and PEG-hybrids.

TABLE S5 Features of micro-complex regions detected in *M. agalactiae* transconjugants and PEG-hybrids.

TABLE S6 List of SNPs detected in micro-complex regions ≤5 nt and their impact in amino acid sequences.

TABLE S7 Sizes of the Homologous Regions (HR) surrounding the PG2-inherited DNA fragments.

TABLE S8 Comparative genome analysis of *M. agalactiae* strains 5632 and 4867 using Breseq tool (Deatherage and Barrick, 2014).

## References

[B1] Arfi Y. Minder L. Di Primo C. Le Roy A. Ebel C. Coquet L. (2016). MIB-MIP is a mycoplasma system that captures and cleaves immunoglobulin G. *Proc. Natl. Acad. Sci. U.S.A*. 113 5406–5411. 10.1073/pnas.1600546113 27114507 PMC4868467

[B2] Ashizawa Y. Yokochi T. Ogata Y. Shobuike Y. Kato J. Ikeda H. (1999). Mechanism of DNA gyrase-mediated illegitimate recombination: characterization of *Escherichia coli* gyrA mutations that confer hyper-recombination phenotype. *J. Mol. Biol.* 289 447–458. 10.1006/jmbi.1999.2758 10356321

[B3] Bailey T. L. Gribskov M. (1998). Combining evidence using p-values: application to sequence homology searches. *Bioinformatics* 14 48–54. 10.1093/bioinformatics/14.1.48 9520501

[B4] Baranowski E. Dordet-Frisoni E. Sagné E. Hygonenq M.-C. Pretre G. Claverol S. (2018). The integrative conjugative element (ICE) of mycoplasma agalactiae: key elements involved in horizontal dissemination and influence of coresident ICEs. *mBio* 9:e873-18 10.1128/mBio.00873-18 PMC6030558 29970462

[B5] Baranowski E. Guiral S. Sagné E. Skapski A. Citti C. (2010). Critical role of dispensable genes in *Mycoplasma agalactiae* interaction with mammalian cells. *Infect. Immun.* 78 1542–1551. 10.1128/IAI.01195-09 20123713 PMC2849427

[B6] Bierne H. Ehrlich S. D. Michel B. (1997). Deletions at stalled replication forks occur by two different pathways. *EMBO J.* 16 3332–3340. 10.1093/emboj/16.11.3332 9214648 PMC1169949

[B7] Blesa A. Baquedano I. Quintáns N. G. Mata C. P. Castón J. R. Berenguer J. (2017). The transjugation machinery of *Thermus thermophilus*: identification of TdtA, an ATPase involved in DNA donation. *PLoS Genet.* 13:e1006669. 10.1371/journal.pgen.1006669 28282376 PMC5365140

[B8] Brigulla M. Wackernagel W. (2010). Molecular aspects of gene transfer and foreign DNA acquisition in prokaryotes with regard to safety issues. *Appl. Microbiol. Biotechnol.* 86 1027–1041. 10.1007/s00253-010-2489-3 20191269

[B9] Budroni S. Siena E. Dunning Hotopp J. C. Seib K. L. Serruto D. Nofroni C. (2011). *Neisseria meningitidis* is structured in clades associated with restriction modification systems that modulate homologous recombination. *Proc. Natl. Acad. Sci. U.S.A.* 108 4494–4499. 10.1073/pnas.1019751108 21368196 PMC3060241

[B10] Calcutt M. J. Lewis M. S. Wise K. S. (2002). Molecular genetic analysis of ICEF, an integrative conjugal element that is present as a repetitive sequence in the chromosome of Mycoplasma fermentans PG18. *J. Bacteriol.* 184 6929–6941. 10.1128/jb.184.24.6929-6941.2002 12446643 PMC135467

[B11] Carver T. J. Rutherford K. M. Berriman M. Rajandream M.-A. Barrell B. G. Parkhill J. (2005). ACT: the artemis comparison tool. *Bioinformatics* 21 3422–3423. 10.1093/bioinformatics/bti553 15976072

[B12] Citti C. Dordet-Frisoni E. Nouvel L. X. Kuo C. H. Baranowski E. (2018). Horizontal gene transfers in mycoplasmas (Mollicutes). *Curr. Issues Mol. Biol.* 29 3–22. 10.21775/cimb.029.003 29648541

[B13] de Vries J. Wackernagel W. (2002). Integration of foreign DNA during natural transformation of *Acinetobacter* sp. by homology-facilitated illegitimate recombination. *Proc. Natl. Acad. Sci. U.S.A.* 99 2094–2099. 10.1073/pnas.042263399 11854504 PMC122324

[B14] Deatherage D. E. Barrick J. E. (2014). Identification of mutations in laboratory-evolved microbes from next-generation sequencing data using breseq. *Methods Mol. Biol.* 1151 165–188. 10.1007/978-1-4939-0554-6_12 24838886 PMC4239701

[B15] Derbyshire K. M. Gray T. A. (2014). Distributive conjugal transfer: new insights into horizontal gene transfer and genetic exchange in mycobacteria. *Microbiol. Spectr.* 2:4 10.1128/microbiolspec.MGM2-0022-2013 PMC4259119 25505644

[B16] Dordet Frisoni E. Marenda M. S. Sagné E. Nouvel L. X. Guérillot R. Glaser P. (2013). ICEA of *Mycoplasma agalactiae*: a new family of self-transmissible integrative elements that confers conjugative properties to the recipient strain. *Mol. Microbiol.* 89 1226–1239. 10.1111/mmi.12341 23888872

[B17] Dordet-Frisoni E. Marenda M. S. Sagné E. Nouvel L. X. Guérillot R. Glaser P. (2013). ICEA of *Mycoplasma agalactiae*: a new family of self-transmissible integrative elements that confers conjugative properties to the recipient strain. *Mol. Microbiol.* 89 1226–1239. 10.1111/mmi.12341 23888872

[B18] Dordet-Frisoni E. Sagné E. Baranowski E. Breton M. Nouvel L. X. Blanchard A. (2014). Chromosomal transfers in mycoplasmas: when minimal genomes go mobile. *mBio* 5:e01958. 10.1128/mBio.01958-14 25425234 PMC4251992

[B19] Faucher M. (2018). *Le Transfert Horizontal de Gènes Chez les Mycoplasmes: de L’acquisition de L’antibiorésistance à la Dynamique Des Génomes.* Master. thesis, University of Toulouse: Toulouse

[B20] Faucher M. Nouvel L.-X. Dordet-Frisoni E. Sagné E. Baranowski E. Hygonenq M.-C. (2019). Mycoplasmas under experimental antimicrobial selection: the unpredicted contribution of horizontal chromosomal transfer. *PLoS Genet.* 15:e1007910. 10.1371/journal.pgen.1007910 30668569 PMC6358093

[B21] Frost L. S. Koraimann G. (2010). Regulation of bacterial conjugation: balancing opportunity with adversity. *Future Microbiol.* 5 1057–1071. 10.2217/fmb.10.70 20632805

[B22] Frost L. S. Leplae R. Summers A. O. Toussaint A. (2005). Mobile genetic elements: the agents of open source evolution. *Nat. Rev. Microbiol.* 3 722–732. 10.1038/nrmicro1235 16138100

[B23] García-Aljaro C. Ballesté E. Muniesa M. (2017). Beyond the canonical strategies of horizontal gene transfer in prokaryotes. *Curr. Opin. Microbiol.* 38 95–105. 10.1016/j.mib.2017.04.011 28600959

[B24] Goessweiner-Mohr N. Arends K. Keller W. Grohmann E. (2014). Conjugation in gram-positive bacteria. *Microbiol. Spectr.* 2:LAS–0004–2013 10.1128/microbiolspec.PLAS-0004-2013 26104193

[B25] Gray T. A. Derbyshire K. M. (2018). Blending genomes: distributive conjugal transfer in mycobacteria, a sexier form of HGT. *Mol. Microbiol.* 108 601–613. 10.1111/mmi.13971 29669186 PMC5997560

[B26] Gray T. A. Krywy J. A. Harold J. Palumbo M. J. Derbyshire K. M. (2013). Distributive conjugal transfer in mycobacteria generates progeny with meiotic-like genome-wide mosaicism, allowing mapping of a mating identity locus. *PLoS Biol.* 11:e1001602. 10.1371/journal.pbio.1001602 23874149 PMC3706393

[B27] Halary S. Leigh J. W. Cheaib B. Lopez P. Bapteste E. (2010). Network analyses structure genetic diversity in independent genetic worlds. *Proc. Natl. Acad. Sci. U.S.A.* 107 127–132. 10.1073/pnas.0908978107 20007769 PMC2806761

[B28] Hörandl E. (2009). A combinational theory for maintenance of sex. *Heredity* 103 445–457. 10.1038/hdy.2009.85 19623209 PMC2854797

[B29] Ikeda H. Moriya K. Matsumoto T. (1981). *In Vitro* Study of illegitimate recombination: involvement of DNA gyrase. *Cold Spring Harbor Symposia Quan. Biol.* 45 399–408. 10.1101/SQB.1981.045.01.054 6271485

[B30] Jackman S. D. Vandervalk B. P. Mohamadi H. Chu J. Yeo S. Hammond S. A. (2017). ABySS 2.0: resource-efficient assembly of large genomes using a bloom filter. *Genom. Res.* 27 768–777. 10.1101/gr.214346.116 28232478 PMC5411771

[B31] Kloesges T. Popa O. Martin W. Dagan T. (2011). Networks of gene sharing among 329 proteobacterial genomes reveal differences in lateral gene transfer frequency at different phylogenetic depths. *Mol. Biol. Evol* 28 1057–1074. 10.1093/molbev/msq297 21059789 PMC3021791

[B32] Kung S. H. Retchless A. C. Kwan J. Y. Almeida R. P. P. (2013). Effects of DNA size on transformation and recombination efficiencies in *Xylella fastidiosa*. *Appl. Environ. Microbiol.* 79 1712–1717. 10.1128/AEM.03525-12 23315739 PMC3591940

[B33] Kurtz S. Phillippy A. Delcher A. L. Smoot M. Shumway M. Antonescu C. (2004). Versatile and open software for comparing large genomes. *Genom. Biol.* 5:R12. 10.1186/gb-2004-5-2-r12 14759262 PMC395750

[B34] Lesic B. Zouine M. Ducos-Galand M. Huon C. Rosso M.-L. Prévost M.-C. (2012). A natural system of chromosome transfer in yersinia pseudotuberculosis. *PLoS Genet.* 8:e1002529. 10.1371/journal.pgen.1002529 22412380 PMC3297565

[B35] Li H. Durbin R. (2009). Fast and accurate short read alignment with burrows-wheeler transform. *Bioinformatics* 25 1754–1760. 10.1093/bioinformatics/btp324 19451168 PMC2705234

[B36] Li H. Handsaker B. Wysoker A. Fennell T. Ruan J. Homer N. (2009). The sequence alignment/map format and SAMtools. *Bioinformatics* 25 2078–2079. 10.1093/bioinformatics/btp352 19505943 PMC2723002

[B37] Lo W.-S. Gasparich G. E. Kuo C.-H. (2018). Convergent evolution among ruminant-pathogenic mycoplasma involved extensive gene content changes. *Genom. Biol. Evol.* 10 2130–2139. 10.1093/gbe/evy172 30102350 PMC6117150

[B38] Marenda M. Barbe V. Gourgues G. Mangenot S. Sagne E. Citti C. (2006). A new integrative conjugative element occurs in Mycoplasma agalactiae as chromosomal and free circular forms. *J. Bacteriol.* 188 4137–4141. 10.1128/JB.00114-06 16707706 PMC1482908

[B39] Meier P. Wackernagel W. (2003). Mechanisms of homology-facilitated illegitimate recombination for foreign DNA acquisition in transformable *Pseudomonas stutzeri*. *Mol. Microbiol.* 48 1107–1118. 10.1046/j.1365-2958.2003.03498.x 12753199

[B40] Mortimer T. D. Pepperell C. S. (2014). Genomic signatures of distributive conjugal transfer among mycobacteria. *Genom. Biol. Evol.* 6 2489–2500. 10.1093/gbe/evu175 25173757 PMC4202316

[B41] Nouvel L.-X. Marenda M. Sirand-Pugnet P. Sagné E. Glew M. Mangenot S. (2009). Occurrence, plasticity, and evolution of the vpma gene family, a genetic system devoted to high-frequency surface variation in Mycoplasma agalactiae. *J. Bacteriol.* 191 4111–4121. 10.1128/JB.00251-09 19376859 PMC2698505

[B42] Nouvel L. X. Sirand-Pugnet P. Marenda M. S. Sagné E. Barbe V. Mangenot S. (2010). Comparative genomic and proteomic analyses of two Mycoplasma agalactiae strains: clues to the macro- and micro-events that are shaping mycoplasma diversity. *BMC Genom.* 11:86. 10.1186/1471-2164-11-86 20122262 PMC2824730

[B43] Ochman H. Lawrence J. G. Groisman E. A. (2000). Lateral gene transfer and the nature of bacterial innovation. *Nature* 405 299–304. 10.1038/35012500 10830951

[B44] Okonechnikov K. Conesa A. García-Alcalde F. (2016). Qualimap 2: advanced multi-sample quality control for high-throughput sequencing data. *Bioinformatics* 32 292–294. 10.1093/bioinformatics/btv566 26428292 PMC4708105

[B45] Pereyre S. Sirand-Pugnet P. Beven L. Charron A. Renaudin H. Barré A. (2009). Life on arginine for *Mycoplasma hominis*: clues from its minimal genome and comparison with other human urogenital mycoplasmas. *PLoS Genet.* 5:e1000677. 10.1371/journal.pgen.1000677 19816563 PMC2751442

[B46] Prudhomme M. Libante V. Claverys J.-P. (2002). Homologous recombination at the border: insertion-deletions and the trapping of foreign DNA in Streptococcus pneumoniae. *Proc. Natl. Acad. Sci. U.S.A.* 99 2100–2105. 10.1073/pnas.032262999 11854505 PMC122325

[B47] Razin S. Hayflick L. (2010). Highlights of mycoplasma research–an historical perspective. *Biologicals* 38 183–190. 10.1016/j.biologicals.2009.11.008 20149687

[B48] Rosengarten R. Behrens A. Stetefeld A. Heller M. Ahrens M. Sachse K. (1994). Antigen heterogeneity among isolates of *Mycoplasma bovis* is generated by high-frequency variation of diverse membrane surface proteins. *Infect Immun*. 62 5066–5074. 7927789 10.1128/iai.62.11.5066-5074.1994 PMC303227

[B49] Rutherford K. Parkhill J. Crook J. Horsnell T. Rice P. Rajandream M. A. (2000). Artemis: sequence visualization and annotation. *Bioinformatics* 16 944–945. 10.1093/bioinformatics/16.10.944 11120685

[B50] Sambrook J. Fritsch E. Maniatis T. (1989). *Molecular Cloning: A Laboratory Manual*, 2nd Edn New York, NY: Cold Spring Harbor.

[B51] Shen P. Huang H. V. (1986). Homologous recombination in *Escherichia coli*: dependence on substrate length and homology. *Genetics* 112 441–457. 3007275 10.1093/genetics/112.3.441 PMC1202756

[B52] Shimizu H. Yamaguchi H. Ashizawa Y. Kohno Y. Asami M. Kato J. (1997). Short-homology-independent illegitimate recombination in *Escherichia coli*: distinct mechanism from short-homology-dependent illegitimate recombination. *J. Mol. Biol.* 266 297–305. 10.1006/jmbi.1996.0794 9047364

[B53] Sirand-Pugnet P. Lartigue C. Marenda M. Jacob D. Barré A. Barbe V. (2007). Being pathogenic, plastic, and sexual while living with a nearly minimal bacterial genome. *PLoS Genet.* 3:e75. 10.1371/journal.pgen.0030075 17511520 PMC1868952

[B54] Smith G. R. (1991). Conjugational recombination in E. coli: myths and mechanisms. *Cell* 64 19–27. 10.1016/0092-8674(91)90205-d 1986865

[B55] Soucy S. M. Huang J. Gogarten J. P. (2015). Horizontal gene transfer: building the web of life. *Nat. Rev. Genet.* 16 472–482. 10.1038/nrg3962 26184597

[B56] Tardy F. Maigre L. Poumarat F. Citti C. (2009). Identification and distribution of genetic markers in three closely related taxa of the *Mycoplasma mycoides* cluster: refining the relative position and boundaries of the Mycoplasma sp. *bovine group* 7 taxon (*Mycoplasma leachii*). *Microbiology* 155 3775–3787. 10.1099/mic.0.030528-0 19696102

[B57] Tardy F. Mick V. Dordet-Frisoni E. Marenda M. S. Sirand-Pugnet P. Blanchard A. (2015). Integrative conjugative elements are widespread in field isolates of *Mycoplasma* species pathogenic for ruminants. *Appl. Environ. Microbiol.* 81 1634–1643. 10.1128/AEM.03723-14 25527550 PMC4325163

[B58] Teachman A. M. French C. T. Yu H. Simmons W. L. Dybvig K. (2002). Gene transfer in *Mycoplasma pulmonis*. *J. Bacteriol.* 184 947–951. 10.1128/jb.184.4.947-951.2002 11807054 PMC134802

[B59] Thorvaldsdóttir H. Robinson J. T. Mesirov J. P. (2013). Integrative genomics viewer (IGV): high-performance genomics data visualization and exploration. *Brief Bioinformatics* 14 178–192. 10.1093/bib/bbs017 22517427 PMC3603213

[B60] Torres-Puig S. Martínez-Torró C. Granero-Moya I. Querol E. Piñol J. Pich O. Q. (2018). Activation of σ20-dependent recombination and horizontal gene transfer in *Mycoplasma genitalium*. *DNA Res.* 25 383–393. 10.1093/dnares/dsy011 29659762 PMC6105099

[B61] Vasconcelos A. T. R. Ferreira H. B. Bizarro C. V. Bonatto S. L. Carvalho M. O. Pinto P. M. (2005). Swine and poultry pathogens: the complete genome sequences of two strains of *Mycoplasma hyopneumoniae* and a strain of Mycoplasma synoviae. *J. Bacteriol.* 187 5568–5577. 10.1128/JB.187.16.5568-5577.2005 16077101 PMC1196056

[B62] Watt A. E. Browning G. F. Legione A. R. Bushell R. N. Stent A. Cutler R. S. (2018). A novel glaesserella sp. isolated from pigs with severe respiratory infections has a mosaic genome with virulence factors putatively acquired by horizontal transfer. *Appl. Environ. Microbiol.* 84 e92–e18. 10.1128/AEM.00092-18 29572210 PMC5960975

[B63] Wollman E. L. Jacob F. Hayes W. (1956). Conjugation and genetic recombination in *Escherichia coli* K-12. *Cold Spring Harb. Symp. Quant. Biol.* 21 141–162. 10.1101/sqb.1956.021.01.012 13433587

[B64] Xiao L. Paralanov V. Glass J. I. Duffy L. B. Robertson J. A. Cassell G. H. (2011). Extensive horizontal gene transfer in ureaplasmas from humans questions the utility of serotyping for diagnostic purposes. *J. Clin. Microbiol.* 49 2818–2826. 10.1128/jcm.00637-11 21697330 PMC3147716

