# Supplementary figures and images for: Mycoplasma Chromosomal Transfer: A Distributive, Conjugative Process Creating an Infinite Variety of Mosaic Genomes
Source: Front Microbiol. 2019 Oct 23;10:2441. doi: 10.3389/fmicb.2019.02441 (PMC6819513; doi:10.3389/fmicb.2019.02441)

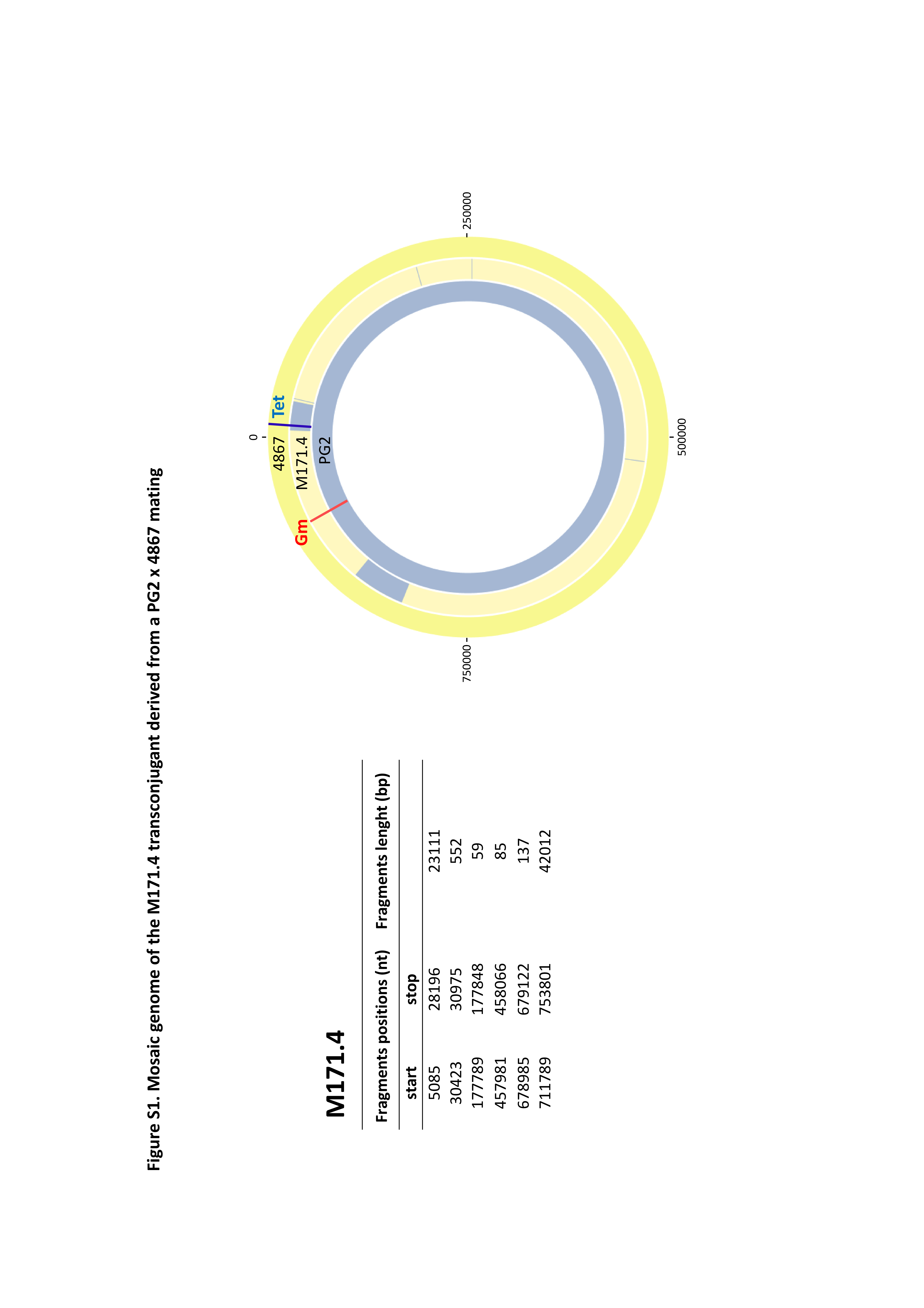

Supplement: FIGURE S1 — Mosaic genome of the M171.4 transconjugant derived from PG2 × 4867 mating. Position and length of PG2 donor fragments present in the M171.4 transconjugant having a 4867 genomic backbone. The DNA plotter depicts the mosaic nature of the M171.4 transconjugant. Chromosomal positions of Gm- (nt-922013) and Tet-markers (nt-20014) are indicated with red and blue line respectively. PG2-specific sequences are color-coded in blue, the 4867 and PG2 genome backbones are depicted in yellow and blue, respectively. From outer to inner circles 4867, M171.4, and PG2. [file Image_1.TIF]

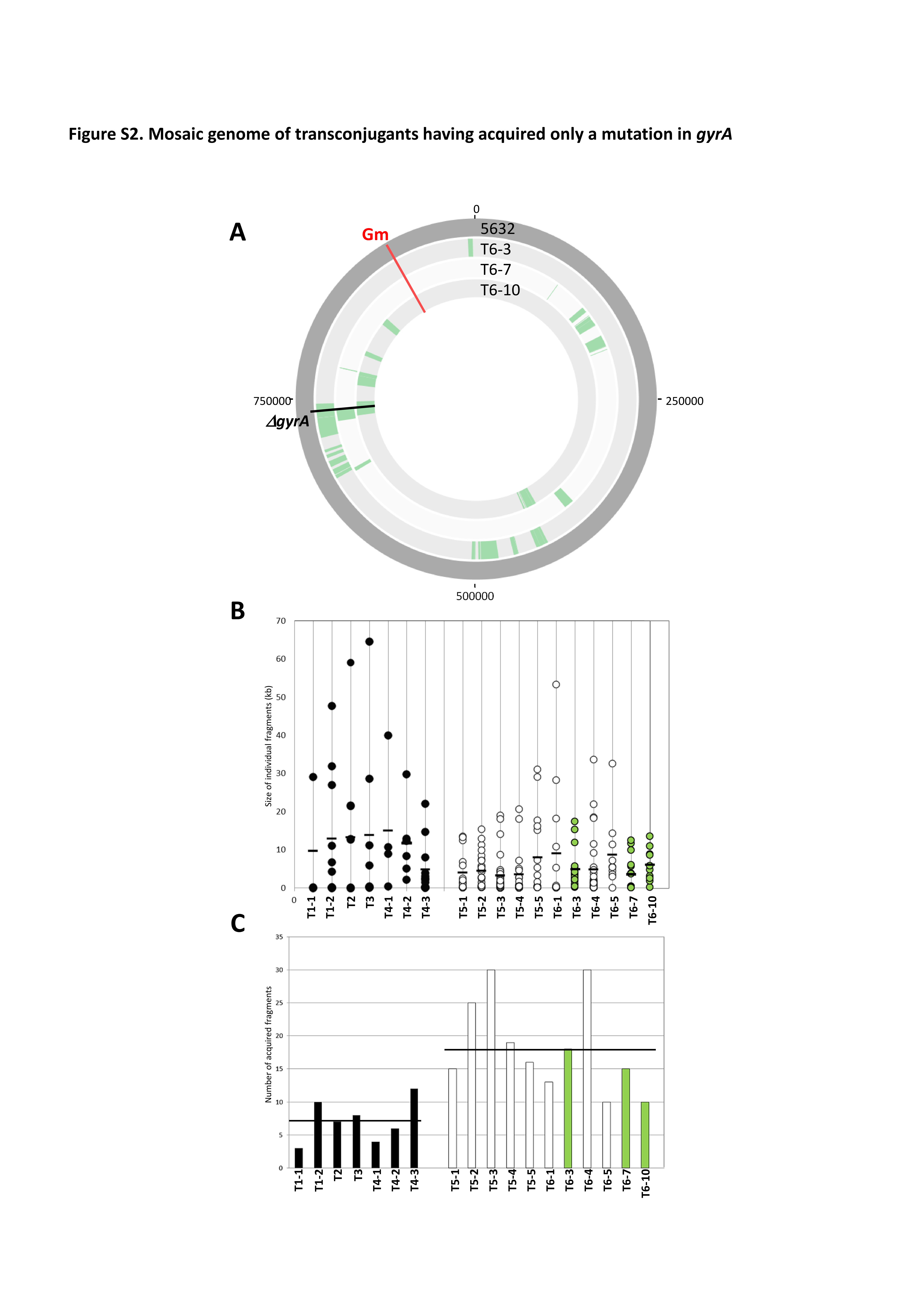

Supplement: FIGURE S2 — Features of EnroS transconjugants having acquired a donor mutated-gyrA but no parE/parC donor. (A) DNA plotter presenting the reconstructed mosaic genomes of transconjugants T6-3, T6-7, and T6-10 that derived from mating M6. These displayed a MIC value of 0.5 μg.mL–1 and were classified as EnroS. Their 5632 genomic backbones are depicted in gray and PG2 specific donor-sequences in green. Chromosomal positions of the 5632-Gm marker (nt-922013) and the acquired PG2 donor gyrA mutation (nt-647997) are indicated by a red and a black line respectively. From outer to inner circles 5632, T6-3, T6-7, and T6-10. (B) Dot plot chart illustrating the number and size of each PG2 fragments incorporated in transconjugants obtained after M1–M6 matings (see Figure 1). Each dot represents a donor-fragment positioned on the graphic depending of its size (kbp). Black thick lines correspond to the mean size of all DNA fragments acquired in individual transconjugants. (C) Bar graph representing the number of PG2 donor fragments acquired by each transconjugants. The average number of acquired DNA fragments is represented by a line. For both graph, (B,C), data obtained with transconjugants produced by single-locus selection matings (see Figure 1; matings M1–M4) are represented by black-dots or bars and those produced by multi-locus selection matings (see Figure 1; matings M5 and M6) by white dots or bars. Transconjugants T6-3, T6-7, and T6-10 are depicted by green dots or bars. [file Image_2.TIF]
