# Supplementary material for: Mycoplasma Chromosomal Transfer: A Distributive, Conjugative Process Creating an Infinite Variety of Mosaic Genomes
Source: Front Microbiol. 2019 Oct 23;10:2441. doi: 10.3389/fmicb.2019.02441 (PMC6819513; doi:10.3389/fmicb.2019.02441)
Supplement: TABLE S2 — Oligonucleotides used in this study. [file Table_2.doc]

| **Primer name** | **Oligonucleotide sequence (5’ -> 3’)** | **products size (bp)** | | | | | | | | |
| --- | --- | --- | --- | --- | --- | --- | --- | --- | --- | --- |
| *M. agalactiae* strains | | **4055** | | **4867** | | **14628** | | **5632** | | **PG2** |
| MAG0220F | CGCCATTGAAGGAACTTGCTCC | 946 | | 946 | | 0 | | 946 | | 0 |
| MAG0220R | TATGCCTGCAGATCAGTCACCA |
| MAGa0900F | ATGGCAATTGATTTACTCCC | 1500 | | 1500 | | 1500 | | 1500 | | 900 |
| MAGa0900R | ATGATGTTGGCCGTATTTGG |
| MAG1560F | GCCGGTGCCCGGGTTAAT | 0 | | 0 | | 0 | | 0 | | 1311 |
| MAG1560R | TCGACACCGCATTTAGCAGCA |
| MAG2450F | AGGACGCTGTAGAGTTTGCAAAGAA | 800 | | 0 | | 0 | | 0 | | 1500 |
| MAG2450R | TACCACTAGCTGCCGCACCA |
| MAGa 3840F | GAAGCACTAAAATCAGGCAA | 1223 | | 1223 | | 1067 | | 1223 | | 1067 |
| MAGa 3840R | GCGGCAGCAGAGTTATCAAG |
| MAGa4400F | CTCTGGCTACAGATTCATCA | 490 | | 490 | | 0 | | 490 | | 607 |
| MAGa4400R | GGTGAACGAGAACAATCGCC |
| MAG5860F | ACCATTGAACTACATTTGCA | 0 | | 494 | | 3000 | | 494 | | 1318 |
| MAGa6510R | AAAACCGCAGACCCAAATGGT |
| MAG5890R | ACATTGCGATGTAGCGGGAACAG |
| II-F1 | TTTATGGCGGTGCAACTGGT | / | | 0 | | / | | 539 | | / |
| Left-1 | TAATGGCCAAGAGTTCAAAAGCAA |
| M8.4_P1 | TTGCCACCCTTATTCATCATC | / | | 0 | | / | | 800 | | / |
| MAGb1260_F | GTAAATGGAGGTGGCAACTTATTCCAATACTTGCTCT |
|  |  |  |  | |  | |  | |  | |

**Table S2.** Oligonucleotides used in the study and products size of identification PCRs
